# Trend in hospitalizations due to ambulatory care-sensitive conditions in the Federal District

**DOI:** 10.1590/0034-7167-2022-0351

**Published:** 2023-05-08

**Authors:** Isabel Pintas Marques Horta, Flávia Reis de Andrade, Lorena da Silva Luz Santos, Naira Pereira de Sousa, Luciano Ramos de Lima, Tania Cristina Morais Santa Bárbara Rehem

**Affiliations:** I Universidade de Brasília. Brasília, Distrito Federal, Brazil

**Keywords:** Primary Health Care, Ambulatory Care Sensitive Conditions, Hospitalization, Health Status Indicators, Health Care Quality, Access, and Evaluation, Atención Primaria de Salud, Condiciones Sensibles a la Atención Ambulatoria, Hospitalización, Indicadores de Salud, Calidad, Acceso y Evaluación de la Atención de Salud, Atenção Primária à Saúde, Condições Sensíveis à Atenção Primária, Hospitalização, Indicador de Saúde, Qualidade, Acesso e Avaliação da Assistência à Saúde

## Abstract

**Objectives:**

to analyze the trend, according to sex, of Ambulatory Care-Sensitive Conditions in the Primary Health Care of the Federal District, from 2009 to 2019.

**Methods:**

ecological trend study using ACSC hospitalization data available in the Hospitalization System of the Single Health System. The *Prais-Winsten* method was used to calculate the annual rate variation, expressed in percentages. The dependent variable was the logarithm of the rates, and the independent one, the years in the time series.

**Results:**

the Federal District registered 2,103,951 general hospitalizations, 16.4% of which were due to Ambulatory Care-Sensitive Conditions. Males had a higher rate of hospitalization in the period, and both sexes showed a stationary trend.

**Conclusions:**

the time trend of Ambulatory Care- Sensitive Conditions was stationary, but further studies are necessary regarding primary health care coverage.

## RESUMO

**Objetivos:**

analisar a tendência das Internações por Condições Sensíveis à Atenção Primária por sexo no Distrito Federal, no período de 2009 a 2019.

**Métodos:**

estudo ecológico de tendência com dados de ICSAP disponíveis no Sistema de Internações Hospitalares do Sistema Único de Saúde. Utilizou-se o método de Prais-Winsten para o cálculo da variação percentual anual das taxas. A variável dependente foi o logaritmo das taxas; e a variável independente, os anos da série temporal.

**Resultados:**

o Distrito Federal registrou 2.103.951 internações gerais, sendo 16,4% Internações por Condições Sensíveis à Atenção Primária. O sexo masculino obteve a maior taxa de internação em todo o período, e houve tendência estacionária tanto para o sexo masculino quanto para o sexo feminino.

**Conclusões:**

as tendências temporais das Internações por Condições Sensíveis à Atenção Primária mostraram-se estacionárias, porém ressalta-se a necessidade de estudos de correlação com a cobertura da Atenção Primária à Saúde.

## RESUMEN

**Objetivos:**

analizar tendencia de Internaciones por Condiciones Sensibles a la Atención Primaria por sexo en Distrito Federal desde 2009 a 2019.

**Métodos:**

estudio ecológico de tendencia con datos de ICSAP disponibles en el Sistema de Internaciones Hospitalarias del Sistema Único de Salud. Utilizado método de *Prais-Winsten* para el cálculo de la variación porcentual anual de las tasas. La variable dependiente fue el logaritmo de las tasas, y la independiente, los años de la serie temporal.

**Resultados:**

Distrito Federal registró 2.103.951 internaciones generales, siendo 16,4% Internaciones por Condiciones Sensibles a la Atención Primaria. El sexo masculino obtuvo la mayor tasa de internación en todo el periodo y hubo tendencia estacionaria tanto para el sexo masculino mientras el femenino.

**Conclusiones:**

las tendencias temporales de las Internaciones por Condiciones Sensibles a la Atención Ambulatoria mostradas estacionarias, pero resaltada la necesidad de estudios de correlación con la cobertura de la atención primaria de salud.

## INTRODUCTION

Primary Health Care (PHC) has had a profound and important impact on political and organizational health care system strategies, especially in the case of universal systems^(1)^. Studies have shown that health systems based on an effective and efficient PHC are associated with better health results^(2-4)^. In this regard, Brazil has been seeking to consolidate its PHC starting with a reorientation of the management model, which is based on the expansion of the Family Health Strategy (ESF)^(5)^. PHC is the level of care that makes use of high-complexity, low-density technologies, being able to deal with more frequent and relevant health issues in its territory when the health system is properly arranged^(6)^. The evaluation of this level of health care contributes for the process of organizing and scheduling actions in the scope of the Single Health System (SUS), allowing a critical reflexion about the quality of work processes^(7)^.

Hospitalizations due to Ambulatory Care-Sensitive Conditions (HACSC) are an indirect indicator of the evaluation and monitoring of the quality of PHC and its availability. This is due to the concept that high HACSC rates are associated with shortcomings in coverage and/or low primary care problem-solving capability. These rates are a warning sign that can trigger analytical mechanisms capable of determining their reasons and potential strategies for them to improve^(8)^. The model proposed by Caminal-Homar & Casanova-Matutano was adapted for the Brazilian situation and published by the Ministry of Health in 2008, including 19 diagnostic groups^(8-9)^. The elaboration of the Brazilian list of sensitive conditions aimed to develop a tool to evaluate primary care and/or use hospital care as a strategy to plan and manage health services, in addition to making it possible to compare performances throughout the national territory^(9)^. Therefore, it is necessary to carry out studies to evaluate the frequency of HACSC in specific territories, to understand the organizational arrangement of PHC and carry out comparative analysis with the different health contexts in Brazil.

## OBJECTIVES

To analyze the trend, according to sex, of Ambulatory Care-Sensitive Conditions in the Primary Health Care of the Federal District, from 2009 to 2019.

## METHODS

### Ethical aspects

Because this research used secondary, public domain data, with no names included, it was not submitted to a Research Ethics Committee.

### Design, period, and place of study

This is an ecological trend analysis, also called time series study^(10)^, guided by the STROBE^(11)^ tool, using data from Ambulatory Care-Sensitive Conditions in the Federal District (FD), according with sex, for all age groups, from 2009 to 2019. The period of the research was chosen as it includes the publication of the Brazilian HACSC list and the latest data made available, during the period of research, by the Hospital Information System from the Single Health System (SIH/SUS).

### Population

The Federal District, which is the Brazilian capital, is in the midwest of the country, and has an estimated population of 3,015,268 people, divided in 33 Administrative Regions. According with the 2019 Administrative Report, the FD was organized in seven health regions, with 174 Primary Health Care Units (UBS) and 433 Family Health Strategy (FHS) teams, with a 44.88% population coverage^(12)^. In the other years, population data from the 2010 Census of the FD were used, as well as intercensus projections, all made available by the Brazilian Institute of Geography and Statistics (IBGE)^(13)^.

### Criteria of inclusion and exclusion

Were included all hospitalizations due to Ambulatory Care-Sensitive Conditions that took place in Brasília, including men and women from any age group. Since hospitalization selection takes place directly in the SIH/SUS, no data needed to be excluded after inclusion criteria were determined.

### Study protocol

The study used data from the Hospitalization System of the Single Health System (SIH/SUS), whose data collection instrument is the Hospitalization Authorization (HA). This system allows categorizing hospitalizations financed by the SUS, and the data are available in the website of the Informatics Department of the Single Health System (DATASUS). ACSC hospitalizations were selected considering which were included in the Brazilian List of Hospitalizations due to Ambulatory Care-Sensitive Conditions^(14)^.

### Analysis of results and statistics

To calculate rate annual percent change (APC), we used the *Prais-Winsten* method, which allows for first degree self-correlation correction. The dependent variable was the logarithm of the rates, and the independent one, the years in the time series. The APC and respective confidence intervals were found using Antunes and Waldman’s formulas^(15)^.

We checked for the occurrence of growing, decreasing, or stable trends for each HACSC group, per sex. The trend was considered stable when the regression coefficient was zero with p > 0.05; decreasing when it was negative with p<0.05; and growing when it was positive with p < 0.05. The trend analysis was carried out using the software *Stata* ^R^, version 13.0.

## RESULTS

**Figure 1:**
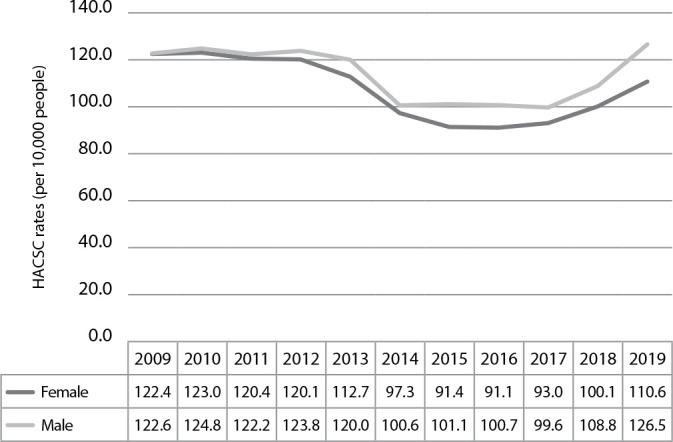
Hospitalizations due to Ambulatory Care-Sensitive Conditions rates in males and females, Federal District, Brazil, 2009-2019

The Federal District registered, from 2009 to 2019, 2,103,951 general hospitalizations, with 344,532 (16.4%) being caused by Ambulatory Care-Sensitive Conditions (ACSC). Considering all groups and causes for hospitalization and diagnostics, the hospitalization rates were higher among males throughout the period analyzed. For both sexes, the highest rates took place in 2010, with 124.78 per 10,000 people for males, and 122.98 per 10,000 people for females (Figure 1).

*HACSC - Hospitalizations due to Ambulatory Care-Sensitive Conditions*

*Source: SIH/SUS, 2021.*

In general, there was a stationary tendency for HACSC in the FD during the period, both for males (APC: -0.91%; CI 95%: -4.51; 2.83) and females (APC: -1.01%; CI 95%: - 4.88; 3.01) (Table 1).

**Table 1:** Trend of Hospitalizations due to Ambulatory Care-Sensitive Conditions rates per group of causes, per sex, Federal District, Brazil, 2009-2019

HACSC	^*^APC %		^†^CI 95%		Interpretation	
	^‡^M	^§^F	M	F	M	F
All HACSC groups	-0.91	-1.01	-4.51; 2.83	-4.88; 3.01	Stationary	Stationary
Diseases preventable through immunization	-2.95	0.20	-5.78; -0.03	-7.66; 8.73	Reduction	Stationary
Infectious gastroenteritis and complications	-0.01	-0.67	-5.81; 6.14	-6.80; 5.86	Stationary	Stationary
Anemia	-13.57	-6.51	-17.63; -9.30	-9.06; -3.89	Reduction	Reduction
Nutritional deficiency	-5.62	-4.31	-8.88; -2.24	-7.55; -0.96	Reduction	Reduction
Ear, nose, and throat infections	4.76	4.26	0.63; 9.06	0.09; 8.61	Increase	Increase
Bacterial pneumonia	0.38	0.80	-6.00; 7.19	-6.15; 8.26	Stationary	Stationary
Asthma	5.36	4.24	0.38; 10.58	-0.97; 9.71	Increase	Stationary
Pulmonary diseases	3.91	2.92	-0.47; 8.48	-2.73; 8.90	Stationary	Stationary
Hypertension	-12.90	-12.58	-17.68; -7.84	-17.36; -7.52	Reduction	Reduction
Angina	-6.08	-7.61	-10.75; -1.18	-11.72; -3.31	Reduction	Reduction
Heart failure	-6.38	-6.73	-8.19; -4.53	-8.59; -4.83	Reduction	Reduction
Cerebrovascular diseases	-6.43	-8.12	-9.19; -3.60	-10.67; -5.50	Reduction	Reduction
*Diabetes mellitus*	-4.71	-6.95	-7.21; -2.14	-9.56; -4.25	Reduction	Reduction
Epilepsy	4.15	4.66	0.20; 8.25	1.46; 7.95	Increase	Increase
Kidney and urinary tract infection	-2.92	-1.64	-4.64; -1.16	-3.11; -0.15	Reduction	Reduction
Skin and subcutaneous tissue infection	-2.41	-2.86	-6.33; 1.67	-6.80; 1.24	Stationary	Stationary
Inflammatory disease in female pelvic organs		-3.07		-6.65; 0.65	Does not apply	Stationary
Gastrointestinal ulcer	-2.62	-2.47	-8.13; 3.23	-9.54; 5.16	Stationary	Stationary
Prenatal and child delivery related diseases	9.79	-1.26	2.61; 17.47	-6.13; 3.85	Increase	Stationary

* HACSC - Hospitalizations due to Ambulatory Care-Sensitive Conditions; APC - annual percent change;

† CI - confidence interval;

‡ M - Male;

§ F - Female.

Comparing the groups of causes of hospitalization in males, we found an increasing trend in the groups of ear, nose, and throat infection (APC: 4.76%; CI 95%: 0.63; 9.06), asthma (APC: 5.36%; CI 95%: 0.38; 10.58), epilepsy (APC: 4.15%; CI 95%: 0.20; 8.25) and prenatal and child delivery-related diseases (APC: 9.79%; CI 95%: 2.61; 17.47). It should be noted that, in males, prenatal and child delivery-related diseases involve congenital syphilis (CID-10 A50) and congenital rubella syndrome (CID 10 P35.0). Therefore, in regard to this group of causes, one must consider the mother-child dyad. On the other hand, there was a relevant decreasing trend for anemia (APC: -13.57%; CI 95%: -17.63; -9.30) and hypertension (APC: -12.90%; CI 95%: -17.68; -7.84). The group of bacterial pneumonia had a stationary trend in the period (APC: 0.38%; CI 95%: -6.00; 7.19).

Regarding females, there was a decreasing trend for nutritional deficiency (APC: -4.31%; CI 95%: -7.55; -0.96), angina (APC: -7.61%; CI 95%: -11.72; -3.31), heart failure (APC: -6.73%; CI 95%: -8.59; -4.83), cerebrovascular disease (APC:-8,12%; CI 95%: -10,67; -5,50), diabetes mellitus (APC: -6.95%; CI 95%: -9.56; -4.25) and kidney and urinary tract infection (APC: -1.64%; CI 95%: -3.11; -0.15); in this regard, the diagnostic groups of anemia (APC: -6.51%; CI 95%: -9.06; -3.89) and hypertension stood out (APC: -12.58%; CI 95%: -17.36; -7.52). There was also a growing trend in the group of ear, nose, and throat infection (APC: -4.26%; CI 95%: 0.09; 8.61) and in the epilepsy group (APC: 4.66%; CI 95%: 1.46; 7.95).

Similarly to the males, infectious gastroenteritis and complications also showed a stationary trend among females (APC: -0.67%; CI 95%: -6.80; 5.86), as did bacterial pneumonia (APC: 0.8%; CI 95%: -6.15; 8.26), pulmonary disease (APC: 2.92%; CI 95%: -2.73; 8.90), skin and subcutaneous tissue infection (APC: -2.86%; CI 95%: -6.80; 1.24), and gastrointestinal ulcer (APC: -2.47%; CI 95%: -9.54; 5.16).

## DISCUSSION

HACSC rates we found in the FD are below those from the states of Minas Gerais (21.12%), according to records from 2008 to 2017, and Rondônia (24.8%), according to records from 2012 to 2016^(16-17)^. The high rates of ACSC hospitalizations and the differences found between states can be interpreted as a deficiency in the quality and problem-solving ability of PHC, especially in regions where ESF is not fully implemented^(18)^. Throughout the period of the study, there were important changes in the FD regarding policies associated with the organization and improvement of PHC. Examples of these actions are decrees from the Ministry of Health that discuss the standardization of work processes in PHC services and the implementation of the National Program for the Improvement in the Availability and Quality of Primary Care (PMAQ), which enforces enhancements to the model of care in the PNAB, the development of workers, and redirects the services to better attend needs of users^(19-21)^.

As a part of the process of consolidation of the PHC, decrees were published in 2016 and 2017 which prioritized and reiterated the choice of the FD health care model considering the ESF. Namely, Resolution No. 465/2016 provided the support for the reform of PHC in the FD, determining that the ESF should be considered as the priority strategy in reorganizing the public health care model^(22)^; in 2017, decrees SES-DF No.77^(23)^ and SES-DF No. 78^(24)^ were created, which, respectively, put into place norms and directives for the FD primary health care policies and specified the rules for the conversion of FD PHC into the ESF model. Although the recent reorganization efforts from the PHC did not lead to a general HACSC reduction, there was a reduction trend in groups of diagnoses concerning the primary health care, such as hypertension and diabetes mellitus. Another study pointed at the reduction in participation of people from 50 to 69 years old in HACSC records, which may lead to improvements in PHC access^(8)^.

Males were more prevalent than females, corroborating findings by Botelho^(25)^ and Macinko^(26)^. Studies showed that there are obstacles to the access of the male population to the health units, including their working hours and the resistance of this population to search for health services, especially those related with PHC, reducing the possibility of providing early treatment. The same is not true for women, who seek these services more often and attempt to prevent these issues^(27)^. Furthermore, complaints and complications related with increasing trends among men are often managed in secondary care, due to the fact that care from specific specialties is usually provided in this level of attention. It should be noted that two diagnoses had the same trend between men and women.

The group of bacterial pneumonia, despite a stationary trend, had a higher HACSC rate in men. A study suggests that risk factors for these causes include old age; comorbidities, especially in the case of chronic conditions; and organism immunodepression^(28)^. In the scope of the SUS, we would like to call attention to a fragmented perspective according to which certain population groups are more vulnerable than others. This is clearly visible in the activities planned for the health of women, children, and the elderly, while those targeted at men’s health are restricted to thematic campaigns or health education^(29)^.

Another factor that contributes to lower HACSC rates in females is the organization of the health system that, throughout the years, has been prioritizing policies and health services targeted at women and children. The attention to some sets of causes of hospitalization was given support from the Ministry of Health, including the Stork Network. Considering the above, we expect the female population to seek health services more often than men, who associate this search with feelings of frailty^(30)^.

The goals of the FD in regard to vaccination coverage are in accordance with the National Immunization Plan (PNI), presenting, from 2010 to 2012, a growing trend, going through a stationary period from 2013 to 2016, and going down from 2017 on. It is important to highlight low coverage of the MMR vaccine (48%) during the measles outbreak in 2019. The abandonment rates of the HPV vaccine among adolescents also stands out, as, from 2013 to 2019, the coverage among girls was only 47.8%. The coverage of vaccines against meningitis C was also lower among girls (43.1%) than among boys (47.2%)^(31)^. Therefore, the behavior, among females, of the set of causes related with diseases that can be prevented through immunization, can reflect the actions prescribed by the PNI to reach vaccine coverage goals, as well as the multiple factors related to the insufficient vaccine coverage in specific age groups.

Regarding hypertension and diabetes mellitus, the trend in the FD in the period was decreasing. These two sets of causes are Noncommunicable Diseases (ND), which are a cause of grave worldwide concern, due to the number of premature deaths and the loss of quality of life. According to a Vigitel research (2019), NDs were responsible for 71% of deaths in Brazil in 2016. In the FD, nearly 28.5% of the population stated to have a diagnosis of hypertension^(32-33)^. Nonetheless, the reduction trend of these diseases may be related with the promotion of effective public policies to prevent and control NDs and their risk factors, coupled with the improvement of health services targeted at chronic care, supported by the Plan of Strategic Action to Combat NDs in Brazil, 2011-2022.

This plan was presented in 2016, by the SES-DF, to be put into effect simultaneously in many fields, to contribute to the control of NDs in the FD^(34)^. Furthermore, the FD showed the highest percentage, among Brazilian states, of healthy dietary habits, with 53.1% of women^(32)^ stating to have diets in line with the guidance from the National Policy of Diet and Nutrition (PNAN) and the District Plan of Nutritional and Food Security (PDSAN). The FD published a public consultation in regard to the District Policy for Food and Nutrition (PDAN), which aims to improve the nutrition and health of its population. This setting shows the sensibility of the district government in guiding their dietary and nutrition actions considering the epidemiological context and the sociodemographic and nutritional situation of its people, which may have contributed for lower rates of hospitalization due to nutritional deficiency in both sexes.

Despite its reducing trend in the period analyzed, kidney and urinary tract infection showed the highest rate of hospitalization due to HACSC among females. This result is in line with studies according to which this type of infection was one of the three greatest causes of HACSC among women. Among other reasons, this is related to anatomic differences between the sexes, since the vagina is in close proximity to the anus, and the female urethra is smaller than the male urethra, contributing for pathogens to be able to enter the female urinary tract^(15,29,35)^.

Infectious gastroenteritis and its complications showed a stationary trend for both sexes. Hospitalizations in this group may result from the lack of access and effectivity of the PHC, but may also be a result of the social structure of the population analyzed, including social determinants such as access to education and housing and financial conditions, which are factors exogenous to PHC^(15)^.

### Study limitations

Ecological studies analyzing historical trends have limitations such as the fact that the aggregate analyses do not control for confounding factors related with problems caused by the quality of the information system that provided the data, including undernotification and categorization errors. Furthermore, the study used the CID-10 codes present in the HA as its database, which can have errors in its registry due to little data in the patients’ records or to the financial interests of the hospital involved^(36)^. The SIH/SUS is still, in Brazil, the largest database related with Single Health System hospitalizations. Thus, its use is recommended for epidemiological analyses to guide health management. It should also be noted that, in 2018, nearly 35.6% of the population of the FD declared to have health insurance, and private sector hospitalizations are not considered in SIH/SUS analyses^(37)^.

### Contributions for the field of nursing, health, and public policy

Studies that use the Brazilian HACSC list contribute to improve and consolidate this indicator in the Brazilian context and are important to plan and formulate policies and strategies to reduce these rates.

## CONCLUSIONS

Time trends of HACSC in the Federal District were stationary. Nonetheless, further correlation studies of primary health care coverage are necessary, especially in regard to recent changes in the health care model. It is also of note the relevance of other factors in the HACSC trends in each territory, such as social health determinants, the process of work of health teams, and the level of support for self-care in the population, all of which have a strong influence on the frequency of each diagnosis. In this regard, the data presented here can help evaluating the quality of care provided in primary health care, indicating where the service can be improved.
